# The proteomics and metabolomics studies of GZU001 on promoting the Merisis of maize (*Zea mays* L.) roots

**DOI:** 10.1186/s12870-023-04130-0

**Published:** 2023-02-21

**Authors:** Zhiguo Zheng, Shunhong Chen, Panpan Wei, Shengxin Guo, Gang Yu, Jian Wu

**Affiliations:** 1 National Key Laboratory of Green Pesticide, Key Laboratory of Green Pesticide and Agricultural Bioengineering, Ministry of Education/Guizhou University, Huaxi District, Guiyang, 550025 People’s Republic of China; 2 The Key Laboratory of Chemistry for Natural Products of Guizhou Province and Chinese Academy of Sciences, Guizhou Provincial Engineering Research Center for Natural Drugs, Guiyang, 550014 China

**Keywords:** Proteomics, Metabolomics, Glucose photosynthesis, Carbohydrate metabolism, Phenylalanine biosynthesis

## Abstract

**Background:**

Plant growth regulators are chemicals that regulate plant growth and development, which can regulate hormonal balance and affect plant growth, thereby increasing crop yield and improving crop quality. Our studies have revealed a new compound, GZU001, which could be used as a plant growth regulator. This compound has been observed to affect root elongation in maize significantly. However, the exact mechanism of this phenomenon is still being investigated.

**Results:**

Metabolomics and proteomics were used in unison in this study to explore the response pathway and regulation mechanism of GZU001 in promoting maize root elongation. From the appearance, we can see that both roots and plants of maize treated with GZU001 are significantly improved. Maize root metabolism revealed 101 differentially abundant proteins and 79 differentially expressed metabolites. The current study identified altered proteins and metabolites associated with physiological and biochemical processes. GZU001 treatment has been demonstrated to promote primary metabolism, essential for carbohydrates, amino acids, energy, and secondary metabolism. The result suggests that the stimulation of primary metabolism is beneficial for the growth and development of maize and plays a significant role in sustaining metabolism and growth.

**Conclusions:**

This study recorded the changes of related proteins and metabolites in maize roots after GZU001 treatment and provided evidence for this compound’s action mode and mechanism in plants.

**Supplementary Information:**

The online version contains supplementary material available at 10.1186/s12870-023-04130-0.

## Background

Maize (*Zea mays* L.) is planted in a large areas around the world and is used as food, feed, and industrial raw materials [1]. Since 2001, maize has been the most fruitful food crop in the world, with an output of 1.134 billion tons (USDA, 2020, https://www.usda.gov/). The success of maize crops largely depends on the root system’s complexity. Roots are essential for the uptake of water and nutrients, their distribution to the aerial parts of plants, supplying energy and anchoring in the soil [2–5]. The root system has a remarkable capacity for adaptation to varying external conditions in terms of development and physiology [6–9]. To optimise maize yield and quality, a deeper understanding of the molecular components that regulate root growth and development is required.

**Fig. 1:**
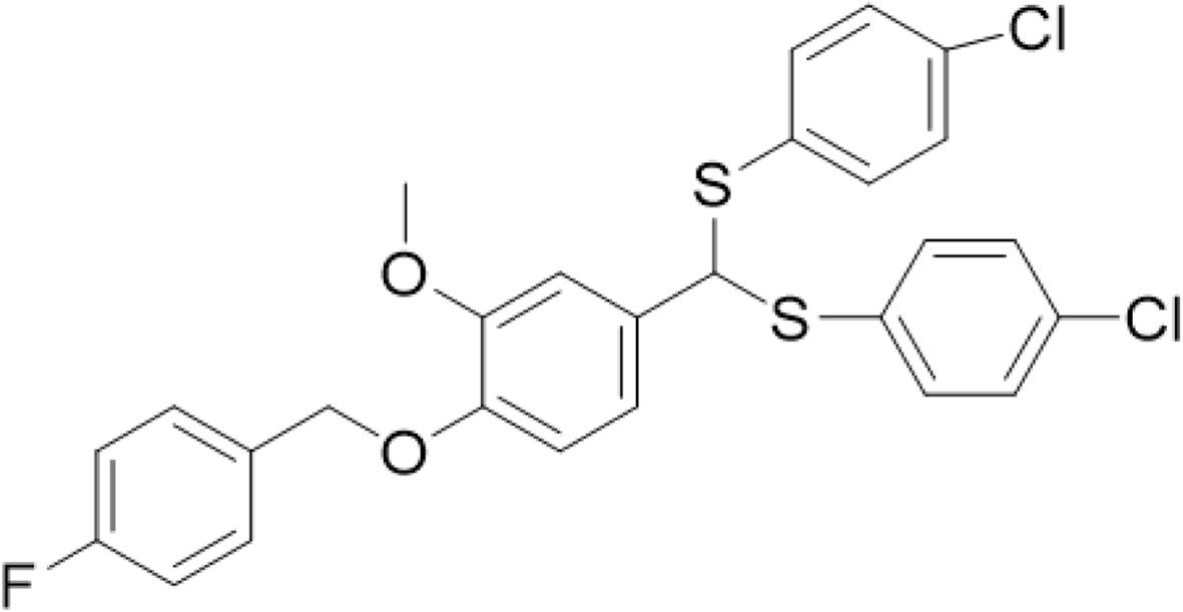
Structure of GZU001

With the modernization of agriculture, it has become very important to ensure high production and financial viability by using chemicals. Regarding plant growth regulation activity, compounds containing aromatic ether structures have been studied extensively. Recently, Hong et al. reported a series of compounds at a concentration of 10 μg/cm^3^ promoting the growth of oilseed rape [10]. The commercially available Zengchanling (4-iodophenoxyacetic acid), 4-chlorophenoxyacetic acid, 2-naphthoxyacetic acid, etc. are all plant growth regulators containing ether structures. They are widely used in agricultural production because of their safety, simple structure, and good biological activity. The previous study synthesized new ether-containing compounds based on natural vanillin and subsequently performed characterization and biological evaluation [11, 12]. GZU001 (see Fig. 1), also named as fubianliusuoyoumi by the National Pesticide Standards Committee of P.R. China, has been found to promote maize root elongation significantly. Data from acute oral toxicity, acute dermal toxicity, chronic inhalation toxicity, skin irritation, acute eye irritation and skin allergy (sensitization) tests have indicated that GZU001 is a low-toxic compound (details can be found in the Supplementary Information Table S6). The exact mechanism of action, however, remains unknown.

People have studied their growth-promoting mechanism more deeply among the wild plant hormones such as auxin and gibberellin. Auxin promotes the cell expansion of hypocotyl cells as one of its functions. It has been determined that the activation of PM H^+^-ATPase and the acidification of the apoplast are responsible for promoting plant cell growth, as suggested by the “acidic growth hypothesis” [13, 14]. The physiological functions of auxin- and GA-responsive genes are not fully elucidated. Still, many of them are thought to participate in regulating cell division cycles and cell wall formation and modification [15].

In recent years, some compound derivatives synthesized by Parus et al., Sumalan et al., and Yang et al. have been considered potential plant growth regulators due to their good growth-promoting effects in the plant growth bioactivity tests [16–18]. In agricultural production, it is necessary to find environmentally benign and inexpensive plant growth regulators. However, the growth-regulating mechanisms of these compounds have rarely been investigated. To assess the capability of GZU001 to regulate plant growth, a preliminary assessment was performed using omics methods, providing a theoretical basis for its subsequent utilization and development.

In the present study, to accurately determine the growth-promoting effect of GZU001 on maize roots, we used a combination of proteomics and metabolomics to characterize the morphological and developmental changes in maize roots treated with GZU001. The metabolites and metabolic pathways were studied, focusing on photosynthesis, carbohydrate metabolism, and phenylpropanoid biosynthesis. These data will provide a basis for the further study of the GZU001 functions in various metabolic pathways to underpin an in-depth analysis of the underlying mechanisms regulating maize root growth.

## Results

### Growth promotion induced by GZU001

**Fig. 2:**
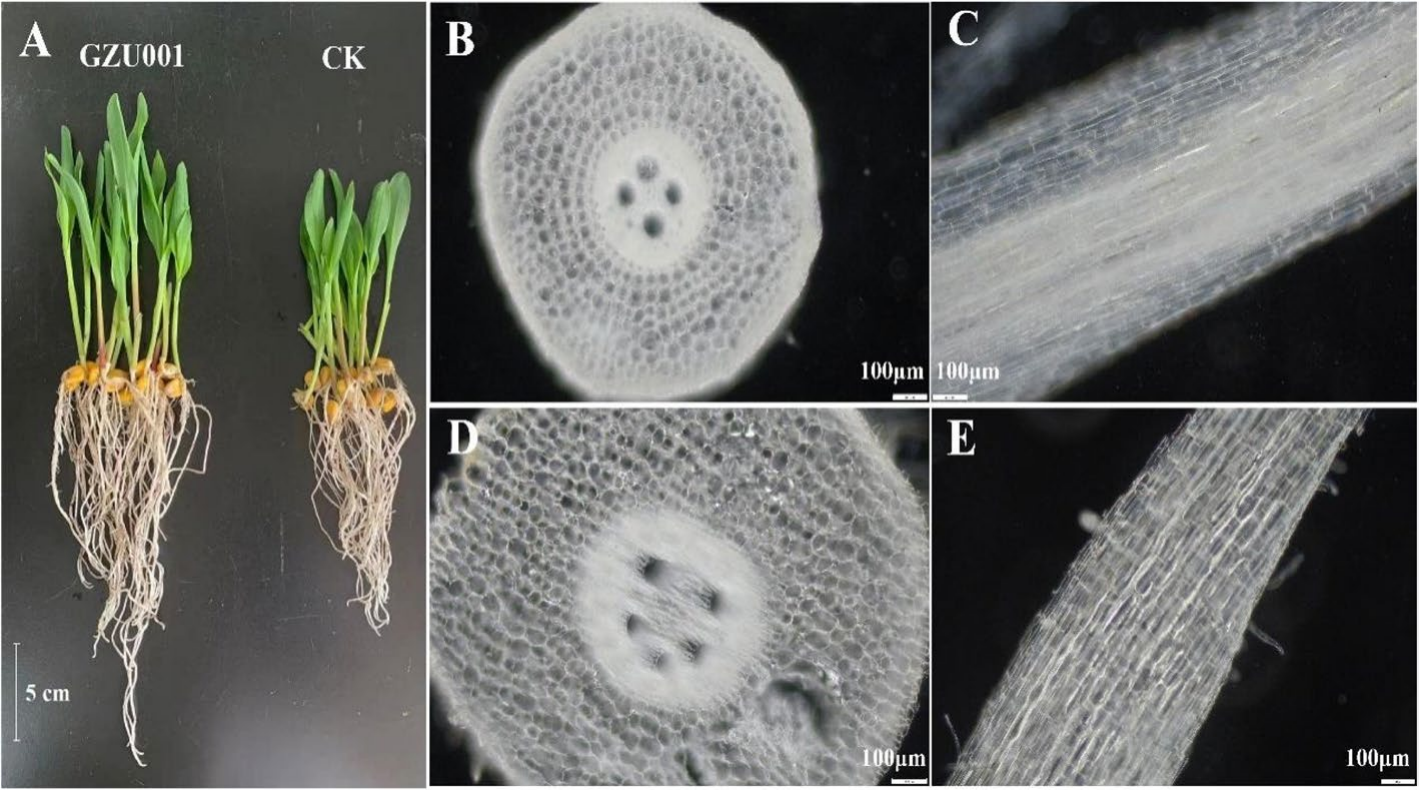
Effects of GZU001 on maize growth. **A**: The growth state of maize; **B**: Cross-section of untreated maize root; **C**: Longitudinal section of the untreated maize root; **D**: Cross-section of maize root treated with GZU001; **E**: Longitudinal section of maize root treated with GZU001

Figure 2 and Table 1 show the results of the growth promotion induced by GZU001. Compared with the control check (CK), the treatment with 0.1 mg/cm^3^ GZU001 promoted maize growth, significantly increasing shoot and root length by 22.81% and 23.86%, respectively (Fig. 2A, Table 1). The current study also observed that maize treated with GZU001 was significantly bigger compared with CK, including root dry weight (62.5%), fresh root weight (54.7%), shoot dry weight (35%), and fresh shoot weight (35.5%). The root system of the plants treated with GZU001 was thicker, indicating that GZU001 positively affected the development of the maize root system. Root slicing experiments showed that the untreated maize root cells (B, C) were neatly and tightly arranged; the GZU001-treated maize root cells (D, E) were disorderly and loosely organized. GZU001 treatment of the maize taproot is correlated with the spatial requirements for root growth. Furthermore, an increased amount of root hairs on the surface of the taproot allows it to absorb more nutrients and sense its surroundings, resulting in an improved plant growth rate.

**Table 1:** Effects of 0.1 g/cm^3^ GZU001 on maize growth parameters

Treatment	Root length (cm)	Shoot length (cm)	Root (4 strains)		Shoot (4 strains)	
			**FW (g)**	**DW (g)**	**FW (g)**	**DW (g)**
CK	13.87 ± 2.18b	11.44 ± 1.95b	1.90 ± 0.26b	0.16 ± 0.02b	2.48 ± 0.53b	0.20 ± 0.04b
GZU001	17.18 ± 2.84a	14.05 ± 1.46a	2.94 ± 0.44a	0.26 ± 0.41a	3.36 ± 0.41a	0.27 ± 0.27a

Means ± SE (*n* = 5). Different letters within a column indicate significant differences at *P* < 0.05 by the *t*-test

*FW* fresh weight, *DW* dry weight

### Protein profiles

**Fig. 3:**
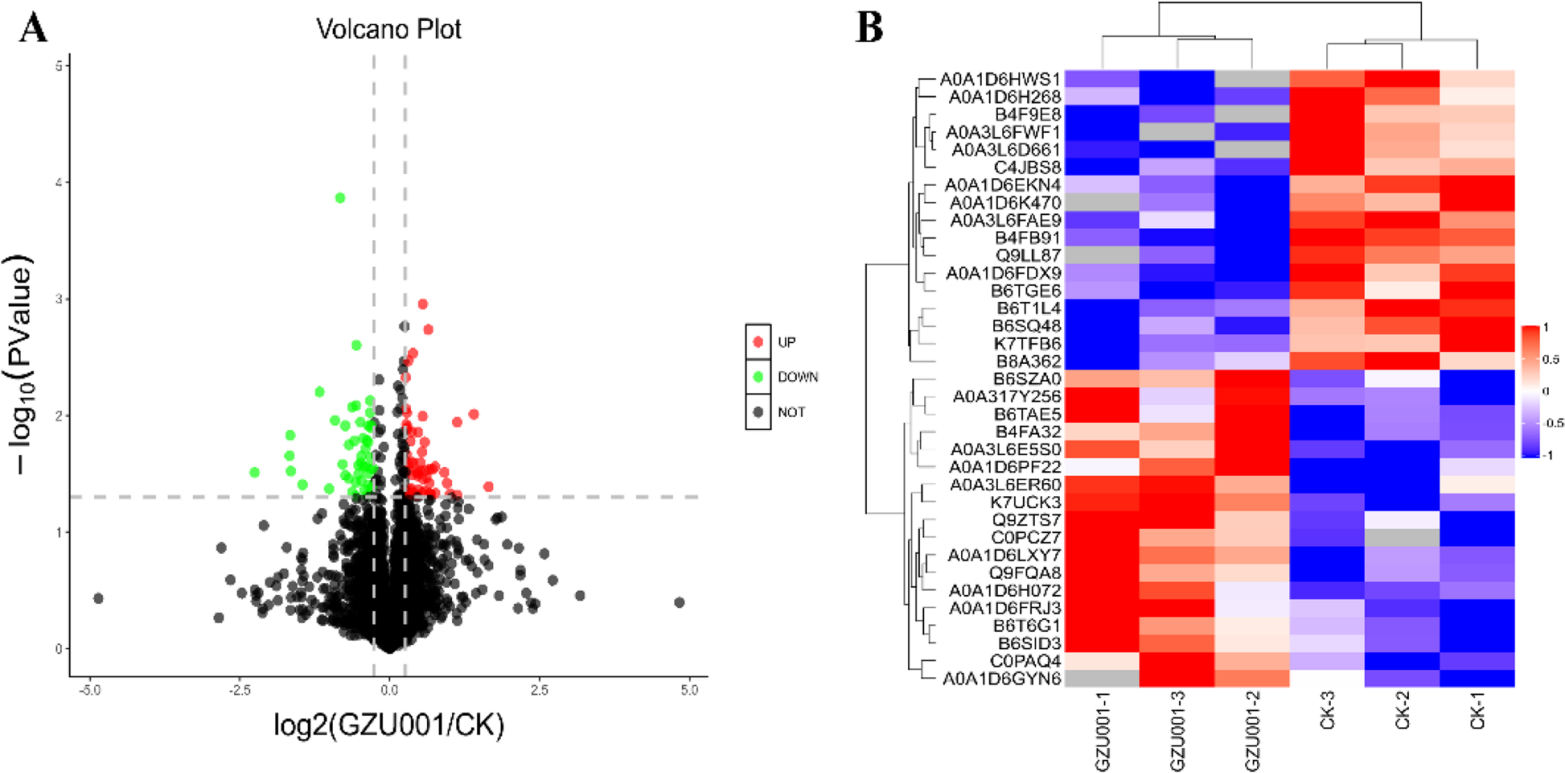
The differentially expressed proteins in the two treatments (FC > 1.5 and *p*-value < 0.05). **A** The volcano graph of the differential protein expression level (GZU001 vs CK). **B** Heatmap of differential protein expression level (GZU001 vs CK). Blue and red colors indicate a decrease and increase, respectively

The present research sought to investigate the molecular effect of GZU001 on maize roots by comparing protein expression and metabolite levels. 24,042 Unique peptides were detected by employing label-free quantitative proteomics. The MASCOT integrated with Proteome Discoverer 1.4 software was employed to identify and annotate 4766 proteins by searching the UniProt database. Using the criteria of fold-change (FC) > 1.5 and *p*-value < 0.05, we identified a total of 35 differentially expressed proteins (DAPs) (Fig. 3A and B). Furthermore, when the same set of samples was used twice or more, 66 DAPs were identified, out of which 56 were higher abundance, and 45 were lower abundance in the GZU0001-treated roots (Table S1). The DAPs were organized into clusters using a hierarchical clustering algorithm and visualized in a heatmap (Fig. 3B).

**Fig. 4:**
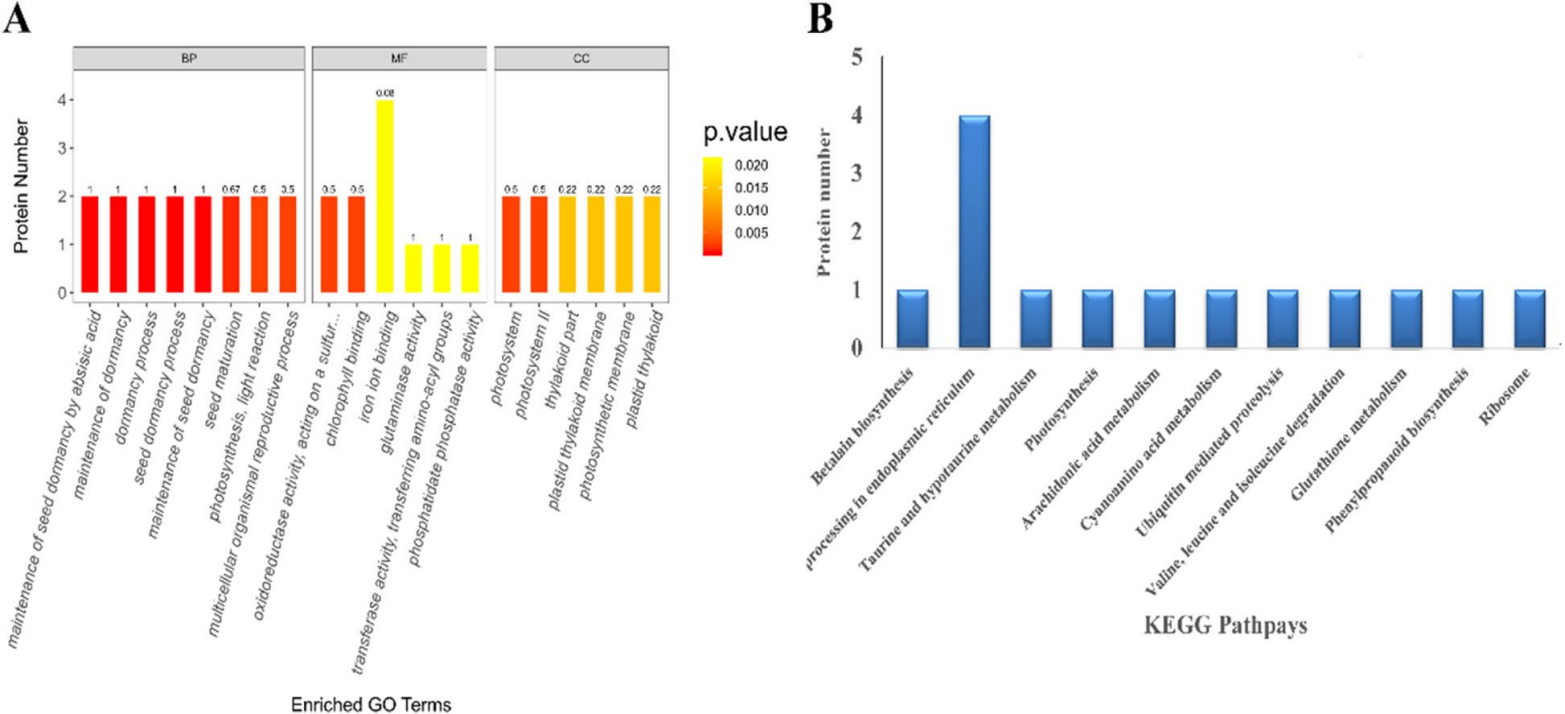
**A** The top 20 most enriched GO terms based on proteomic analysis of GZU001 vs CK. **B** Identified as the KEGG pathway involved in proteins that promote maize root growth

Based on their functional features, 101 DAPs were classified into the biological process (BP), cellular component (CC), and molecular function (MF) (Fig. 4A, Table S2). Significant changes in the BP terms were mainly associated with seed dormancy and reproduction of organisms; the main MF terms were related to catalytic activity, binding activity and transporter activity; and the CC terms included photosynthesis-related organelles and cell membranes.

Using Fisher’s exact test, we employed KEGG analysis to characterize the molecular pathways of DAPs. The primary sites of DAPs are metabolic pathways, including the endoplasmic reticulum, cyanoamino acid metabolism, photosynthesis, valine, leucine, isoleucine degradation, arachidonic acid metabolism, glutathione metabolism, taurine and hypotaurine metabolism, betalain biosynthesis, ribosome, phenylpropanoid biosynthesis, and ubiquitin-mediated proteolysis (Fig. 4B).

### Parallel reaction monitoring (PRM) verification

To ensure the accuracy of our results, nine DAPs were chosen for a PRM assay, including three that were up-regulated and five down-regulated. The results showed that their expression trends remained consistent between label-free and PRM assays (Table 2), indicating the reliability of the label-free method used in this study.

**Table 2:** PRM verification of the expression quantities of particle proteins

Protein	Protein name	PRM results	Label-free results	
		**GZU001/CK**	**GZU001/CK**	***P*-value**
A0A1D6HWS1	Dirigent protein	0.889241	0.313711	0.022159
A0A3L6EKC7	Peroxidase	1.177471	up	-
B4F9E8	17.4 kDa class III heat shock protein	0.44569836	0.647749	0.045259
B4FA32	Peroxidase	2.006250	1.638605	0.028904
B6U8P6	ABA-responsive protein	0.541998	down	-
K7TFB6	ABA-responsive protein	0.851799	0.622959	0.034691
K7VD78	Putative chaperone clbp family protein	0.714123	down	-
Q9ZTS7	Peroxidase	1.053833	1.513926	0.029444

### Metabolite profiles

An exploration was conducted to assess the effects of DAPs on maize’s physiological processes and metabolic activities. Furthermore, the metabolites of maize roots were quantitatively compared. The changes in the metabolic profiles were analyzed by the metabolomics method. Peaks extracted from all the experimental and quality control samples were subjected to Pareto-scaling for multivariate analysis. OPLS-DA, PLS-DA, and PCA revealed a significant distinction between the two treatments (Fig. S1), indicating that GZU001 significantly affected metabolites.

**Fig. 5:**
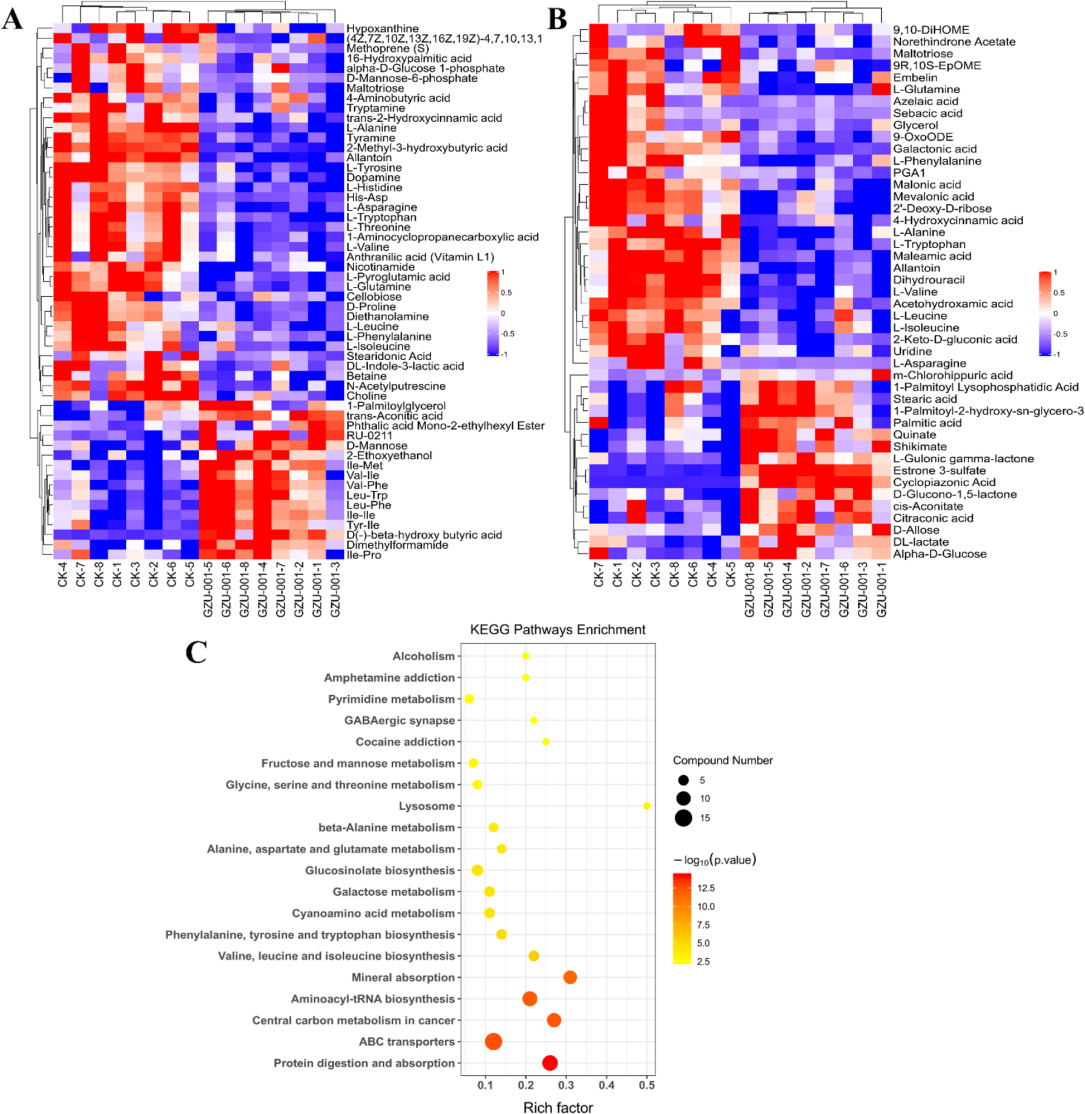
Root metabolic profiles in the GZU001-treated maize. Heatmap of the differential abundant metabolites in the positive mode (**A**) and negative mode (**B**). Each square and color indicate the fold change value of a differentially changed protein. Blue and red colors indicate a decrease and increase in fold change values compared with untreated CK; **C** Pathway analysis of GZU001 vs CK

The metabolic profiles were assessed by detecting 9841 components in the positive and 6864 components in the negative ion modes. Based on the OPLS-DA model with restrictions VIP > 1 and *p*-value < 0.05, 89 metabolites showed significant changes under positive and negative ions modes. Among these, 27 metabolites were higher abundance, and 52 were lower abundance after the treatment with GZU001 (Table S3). Most of these metabolites were lipids, carbohydrates, organic acids, purines, and amino acids. The current study performed univariate analyses to assess the and significance of various differentially expressed metabolites by utilizing the volcano plot integrated fold-change analysis and the *t*-tests (Fig. S2). Additionally, hierarchical clustering calculation, according to the differences in the expression of metabolites in the two treatments. It was also helpful in the choosing of marker metabolites as well as in studying the variations of the relevant processes (Fig. 5A and B).

We used the KEGG website to investigate the pathways related to the differentially expressed metabolites. They were mainly involved in secondary metabolite biosynthesis, sugar metabolism, amino acid biosynthesis, and phenylpropanoid biosynthesis (Fig. 5C). The study results provide essential information on the effects of GZU001 in promoting maize root elongation.

## Discussion

**Fig. 6:**
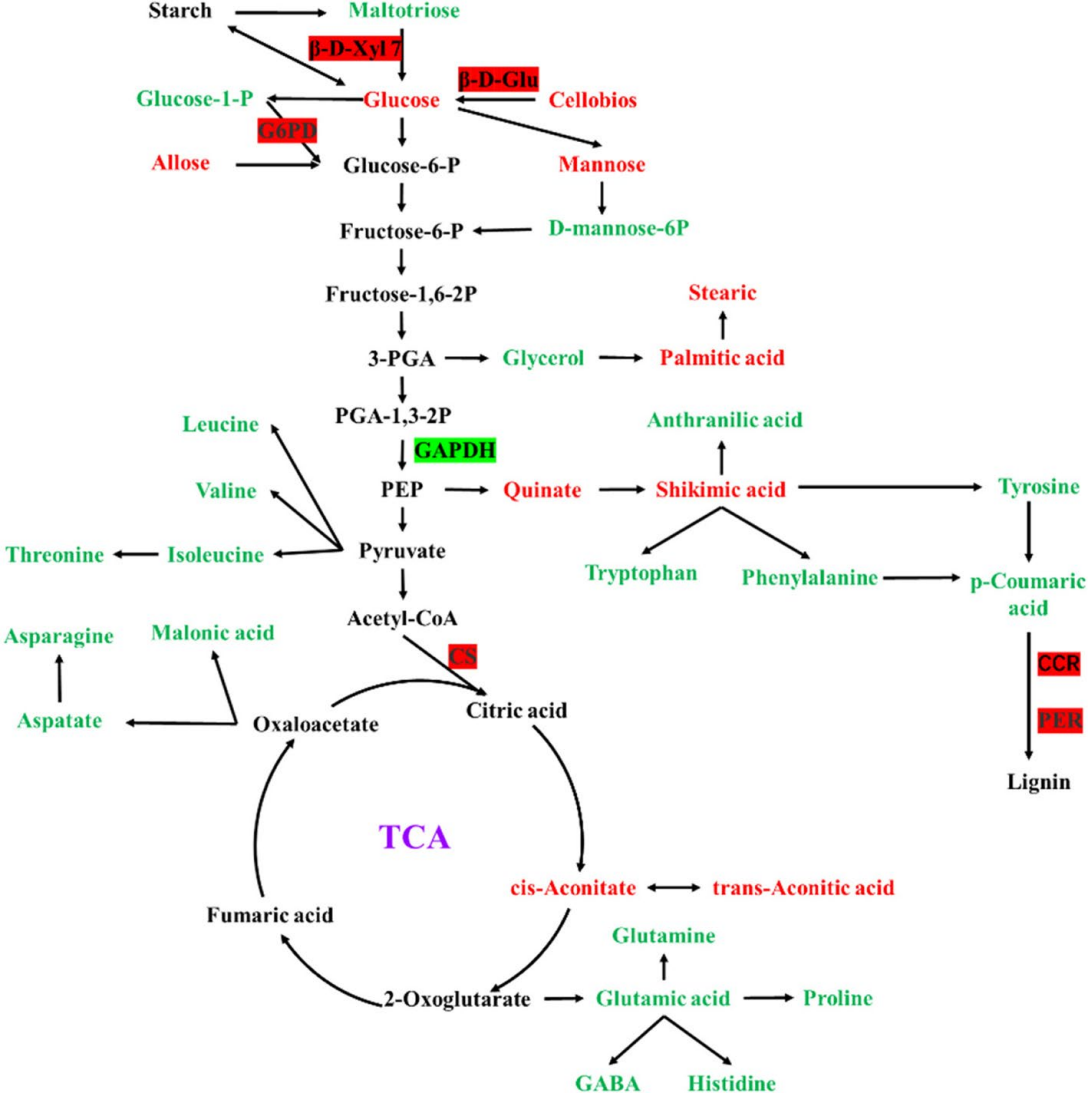
Graphical depiction of metabolite and protein fluctuations. Red indicates upregulation; green indicates downregulation. β-D-Xyl7, beta-D-xylosidase 7; β-D-Glu, beta-D-glucosidase; G6PD, glucose-6-phosphate-1-dehydrogenase; GAPDH, glyceraldehyde-3-phosphate dehydrogenase; CS, citrate synthase; CCR, cinnamoyl-CoA reductase 1; PER, peroxidase

As mentioned before, carbohydrates are precursors of many substances in the metabolism and play a crucial role in energy balance in living organisms. The glycolytic pathway is a complex metabolic network that integrates amino acid biosynthesis, the tricarboxylic acid (TCA) cycle, carbohydrate metabolism, and phytohormone regulation (Fig. 6). Examining these data can help us to understand better the part GZU001 plays in stimulating plant growth.

Carbohydrate metabolism uses the captured photosynthetic energy to provide plants with the carbon they need, which is essential for producing new tissue. Recent research has demonstrated that glucose not only functions as an energy source and a structural element in plant metabolism but also participates in many plant’s metabolic processes as a signal molecule, influencing physiological and biochemical processes such as seed germination, hypocotyl elongation, cotyledon extension, root growth and development, flowering, and senescence [19, 20]. This study revealed that three proteins, including glucose translocator 1 (A0A1D6FDX9), putative beta-D-xylosidase7 (A0A3L6FXJ8), and beta-D-glucosidase (Q53WW9), which are involved in the glycolytic pathway, were significantly up-regulated (Fig. 6, Table S4). The glucose content also increased as expected, so the current study showed that the glucose synthesis pathway was enhanced after treatment with GZU001. As expected, two essential proteins involved in PPP and TCA, including glucose-6-phosphate-1-dehydrogenase (A0A3L6ER60) and citrate synthase (B6TAE5), were significantly up-regulated, which may promote the decomposition of glucose to provide energy and structural components for plant growth and development (Fig. 6, Table S4).

The sensor Target of Rapamycin (TOR) is the main regulatory target. As an upstream regulator of TOR, sugar participates in plant metabolism, transcription, and translation to regulate cell proliferation, growth, and development [21–23]. Previous studies reported that TOR transduces photosynthesis-derived glucose energy signals, thereby controlling the proliferation of stem/progenitor cells in the root meristem [24]. Previous studies [9, 25–28] prove that glucose as a signalling molecule may regulate many critical metabolic processes and activation pathways.

Evidence has been presented to indicate that sugar metabolism can provide the energy required for amino acid metabolism [29]. In plants, amino acids are the precursors of a variety of hormones, and auxin regulates the growth and development of plants in many aspects, such as seed germination, root structure and leaf formation [30]. Auxin biosynthesis correlates with the sugar levels produced and transported to roots [5, 31]. Recently, it has been reported that cytochrome P450 can convert tryptophan (Trp) to indole-3-acetaldoxime (IAAOx) in vitro, which is a key intermediate in IAA biosynthesis in vivo [32, 33]. Auxin-binding protein 4 (ABP4) participates in the growth of maize seedlings, mediates the response of seedlings to auxin, and interacts with light signal pathways [34]. In the current study, a significant decrease in ABA-responsive protein content and an increase in ABP4 and cytochrome P450 (Fig. 6, Table S5) may play a vital role in the development of maize roots.

Plants can transform phenylalanine into basic phenolic compounds, which are combined to create lignin—a vital cell wall component. It is well known that a reduction in lignification and an increase in cell wall relaxation help reduce the stiffness of the cell wall and promote root elongation. Notably, It was observed that the phenylalanine biosynthesis pathway involved in lignin synthesis had a decreased concentration of phenylalanine and tyrosine. Additionally, there was an increase in the abundance of peroxidase (PER, E.C.1.11.1.7) and cinnamoyl-CoA reductase 1 (CCR, E.C.1.2.1.44) (Fig. 6, Table S5). CCR is one of the critical proteins involved in lignin synthesis, and it can catalyze the conversion of three hydroxyl cinnamyl CoA into cinnamaldehyde by NADPH [35]. The synthesis and relaxation of the cell wall are essential in the process of cell elongation [36–39]. In the current study, proteins involved in cell wall relaxation and repair, such as β-D-glucosidase, glycine-rich cell wall structural protein, dirigent protein, putative beta-D-xylosidase7 protein, and expansin protein, had altered activities (Table S5). The softening of the cell wall structure provides enough space for cell expansion, similar to how auxin promotes cell growth. These data (Fig. 6, Table S5) are consistent with the experimental results of root slices (Fig. 2, Table 1), and GZU001 may soften and relax the cell wall structure of maize roots, providing favourable conditions for cell expansion and root growth.

## Conclusion

The proteomic and metabolomic analysis of maize treated with GZU001 provided important information on the mechanism of growth promotion. The DAPs found were mainly involved in carbohydrate metabolism, amino acid metabolism, energy metabolism and secondary metabolism, indicating that the enhancement of primary metabolism after GZU001 treatment may play a certain role in the growth and development of maize. These findings provide a basis for revealing maize growth and development’s molecular mechanisms, but further studies are still needed. The results laid a foundation for further research on the role of GZU001 in regulating DAPs in maize.

## Materials and methods

### Pareparation of GZU001

The compound GZU001 was obtained through a two-stage reaction, beginning with vanillin as the starting material. These stages involved an etherification reaction with 4-fluorobenzyl chloride and a condensation reaction with 4-chlorothiophe nol. The chemical structure of GZU001 was ascertained by nuclear magnetic resonance (NMR) spectroscopy and high-resolution mass spectrometry (HR-MS). More details could be found in a previous article of our laboratory [11, 12].

### Maize cultivation and growth measurements

The maize used as the experimental material was Zhengdan 958, a summer maize hybrid widely grown in China with a growth cycle of about 3–4 months, purchased from Henan Dacheng Seeds Industry Co., Ltd. The preliminary experiments indicated GZU001 had the most obvious effect on the elongation of maize roots at the concentration of 0.1 mg/cm^3^ [11, 12]. The maize seeds were divided into CK group and the GZU001 group randomly. Seeds were soaked in 0.1 mg/cm^3^ of GZU001 for the treatment, whereas distilled water was used for the CK group. After 12 h, maize seeds were placed in square pots (four seeds/pot). They were grown in a greenhouse at 26–28 °C and 70% relative humidity with a 14:10 light cycle at Guizhou University (GZU), Guiyang, China (26°25’N, 106°40’E, 1090 m a.s.l.), the experimental period is from February to March 2020.

The seeds were germinated in pots and cultivated for 6 days (Fig. 2), followed by washing with double distilled water, blotting with paper towels, and growing into roots and shoots. A ruler measured the size of the shoot and the root. After determining the fresh weight of roots and shoots of all four plants in each experimental unit, plant samples were dried to constant weight, and the dry weight was measured. The root system at the root apex was chosen for sectioning by utilizing the free-hand slice method. Subsequently, the tissue cells of the root were viewed through an optical microscope (BX60; Olympus, Tokyo, Japan) and captured with a microscope camera system. The fresh root samples were preserved at -80 °C.

### Label-free analysis of proteins

According to previous studies, protein extraction, digestion, LC–MS/MS, and data analysis were carried out [40, 41]. Proteins were extracted from maize root samples by lysis using 0.1 M DTT, 4% (w/v) SDT, SDS, and 0.1 M Tris–HCl at pH 7.6 [42]. Then, the samples were digested with trypsin according to the filter-aided sample preparation (FASP) method [43]. To analyze each fraction, nano LC–MS/MS was utilized. Further information on the procedure is available in Additional file 1. The data obtained have been uploaded to the Proteome Xchange database (PXD026760).

### Untargeted metabolomics analysis

After grinding samples in liquid nitrogen, 1 cm^3^ of acetonitrile: methanol: water solvent (2:2:1, v/v) was added in 0.06 g of the sample and vortexed for 60 s. After sonication twice at low temperature for 0.5 h, the extracts were left at -20 °C for 1 h to precipitate the protein [44]. Afterward, they were filtered through a filter tube at 14,000 rcf and centrifuged at 4 °C for 20 min; the supernatant was freeze-dried and stored at -80 °C. LC/MS analyses were performed using a UHPLC (1290 Infinity LC, Agilent Technologies) coupled to a quadrupole time-of-flight mass spectrometer (AB Sciex Triple TOF 6600) in Shanghai Applied Protein Technology Co., Ltd. The raw MS data (wiff. scan files) were converted to MzXML files using Proteo Wizard MS Convert before importing them into freely available XCMS software [45, 46]. Detailed procedures are available in Additional file 1.

### Bioinformatics analysis

The current study used Blast2Go (https://www.blast2go.com/) [47] software for GO functional annotation of all proteins identified. The Fisher’s exact test was performed for GO functional enrichment analysis of DAPs, KAAS software was used for KEGG and Genomes pathway annotation of the target proteins. The metabolites were blasted against the online KEGG database to retrieve their GOs and were subsequently mapped to pathways in KEGG [48].

### Statistical analysis

The statistical difference was tested by analysis of variance, and the average difference was compared using the *t*-test (*P* < 0.05). In the parameter measurement of maize growth, 20 maize plants were grown. When measuring root and stem length, the data presented are the average of 20 biological replicates; in measuring the dry and fresh weights of root and shoot, there were 5 biological replicates, and each replicate was the average of the total weight of each 4 maize plants. Statistical analyses were done using SPSS (version 26.0, IBM, Armonk, USA).

## Supplementary Information

**Additional file 1: Fig. S1.** Scores plots of principal components 1 (t[1]) and 2 (t[2]) of the PCA, PLS-DA, OPLS-DA results from maize roots at different treatment. **Fig. S2.** Root metabolic profiles after adding GZU001.

**Additional file 2: Table S1.** Identification of Significantly different modulated metabolites by Label-free proteome analysis in the roots of maize. **Table S2.** GO enrichment analysis of differentially modulated proteins. **Table S3.** Identification of Significantly different modulated proteins by UHPLC Q-TOF MS technology analysis in the roots of maize. **Table S4.** Carbohydrate metabolism-related proteins and metabolites. **Table S5.** Amino acid metabolism pathway and phenylalanine synthesis. **Table S6.** Safety testing for GZU001.

## Data Availability

The mass spectrometry proteomics data have been deposited to the Proteome Xchange Consortium via the PRIDE partner repository with the dataset identifier PXD026760 (http://proteomecentral.proteomexchange.org/cgi/GetDataset?ID=PXD026760). The raw metabolomics data is stored in MetaboLights (https://www.ebi.ac.uk/metabolights/); its dataset identifier is MTBLS5933. All relevant data can be found within the manuscript and its supporting materials.

## Abbreviations

DAPs: Differentially abundant proteins
CK: Control check
FW: Fresh weight
DW: Dry weight
FC: Fold-change
BP: Biological process
CC: Cellular component
MF: Molecular function
PRM: Parallel reaction monitoring
TCA: Tricarboxylic acid
β-D-Xyl7: Beta-D-xylosidase 7
β-D-Glu: Beta-D-glucosidase
G6PD: Glucose-6-phosphate-1-dehydrogenase
GAPDH: Glyceraldehyde-3-phosphate dehydrogenase
CS: Citrate synthase
CCR: Cinnamoyl-CoA reductase 1
PER: Peroxidase
TOR: Target of Rapamycin
Trp: Tryptophan
IAAOx: Indole-3-acetaldoxime
ABP4: Auxin-binding protein 4
GZU: Guizhou University
FASP: Filter-aided sample preparation
NMR: Nuclear magnetic resonance
HR-MS: High-resolution mass spectrometry
