# Supplementary material for: The proteomics and metabolomics studies of GZU001 on promoting the Merisis of maize (Zea mays L.) roots
Source: BMC Plant Biol. 2023 Feb 21;23:103. doi: 10.1186/s12870-023-04130-0 (PMC9942296; doi:10.1186/s12870-023-04130-0)

Supplementary materials

Materials and methods

1.1 Label-free analysis methods

Protein Extraction(Thiellement *et al.*, 2007). Protein was extracted from maize root samples by lysis using SDT (4% (w/v) SDS, 0.1M DTT, 100mM Tris/HCl pH 7.6). Briefly, the samples were frozen in liquid nitrogen and ground with a pestle and mortar. 5 times the volume of TCA/acetone (1:9) was added to the powder and mixed by a vortex. The mixture was placed at -20°C for 4 h and centrifuged at 6000 g for 40 min at 4°C. The supernatant was discarded. The pre-cooling acetone was added and washed three times. The precipitation was air-dried. 30 times the volume of SDT buffer was added to 20-30 mg powder, mixed and boiled for 5 min. The lysate was sonicated and then boiled for 15 min. After centrifuging at 14000 g for 40 minutes, the supernatant was filtered with 0.22 μm filters. The filtrate was quantified with the BCA Protein Assay Kit (Bio-Rad, USA).

*Filter-aided sample preparation (*Wiśniewski *et al.,* 2009*)*. The peptides of each sample were desalted on C18 Cartridges (Empore™ SPE Cartridges C18 (standard density), bed I.D. 7 mm, volume 3 cm^3, Sigma), concentrated by vacuum centrifugation and reconstituted in 40 mm^3 of 0.1% (v/v) formic acid. The peptide content was estimated by UV light spectral density at 280 nm using an extinction coefficient of 1.1 of 0.1% (g/ dm^3) solution that was calculated based on the frequency of tryptophan and tyrosine in vertebrates proteins.

Mass Spectrometry. The peptide mixture was loaded onto a reverse-phase trap column（Thermo Scientific Acclaim PepMap100, 100μm*2cm, nano Viper C18）connected to the C18 reversed-phase analytical column (Thermo Scientific Easy Column, 10 cm long, 75 μm inner diameter, 3 μm resin) in buffer A (0.1% Formic acid) and separated with a linear gradient of buffer B (84% acetonitrile and 0.1% Formic acid) at a flow rate of 300 nL/min controlled by IntelliFlow technology. LC-MS/MS analysis was performed on a Q Exactive mass spectrometer (Thermo Scientific) coupled to Easy nLC (Proxeon Biosystems, now Thermo Fisher Scientific). The mass spectrometer was operated in positive ion mode. MS data were acquired using a data-dependent top10 method, dynamically choosing the most abundant precursor ions from the survey scan (300–1800 m/z) for HCD fragmentation. The automatic gain control (AGC) target was set to 1e6, and the maximum injection time to 10 ms. Dynamic exclusion duration was 40.0 s. The survey scans were conducted at 70,000 m/z 200 resolution, and the resolution for the HCD spectra was established at 17,500 m/z 200, along with an isolation width of 2 m/z. The normalized collision energy was 30 eV, and the underfill ratio, which specifies the minimum percentage of the target value likely to be reached at maximum fill time, was defined as 0.1%. The instrument was run with peptide recognition mode enabled. The MS data were analyzed using Max Quant software version 1.5.3.17 (Max Planck Institute of Biochemistry in Martinsried, Germany)(Cox and Mann, 2008).

1.2 Untargeted metabolomics analysis methods

*LC/MS analysis(*Choi *et al., 2020)*. The ESI source conditions were set as

follows: Ion Source Gas (Gas1, Gas2) as 60, curtain gas (CUR) as 30, source temperature: 600°C, Ion Spray Voltage Floating (ISVF) ± 5500 V. In MS only acquisition, the instrument was set to acquire over the m/z range 60-1000 Da, and the accumulation time for TOF MS scan was set at 0.20 s/spectra. In auto MS/MS acquisition, the instrument was set to acquire over the m/z range of 25-1000 Da, and the accumulation time for the production scan was set at 0.05 s/spectra. The production scan is acquired using information-dependent acquisition (IDA) with high sensitivity mode selected. The parameters were set as follows: the collision energy (CE) was fixed at 35 V with ± 15 eV; declustering potential (DP), 60 V (+) and −60 V (−); exclude isotopes within 4 Da, candidate ions to monitor per cycle: 10.

Data Analysis and statistical data analysis(Fan *et al.*, 2018). Compound identification of metabolites by MS/MS spectra with an in-house database established with available authentic standards. After normalizing the processed data to total peak intensity, we uploaded it to SIMCA-P (version 14.1, Umetrics, Umea, Sweden) for multivariate data analysis. This analysis included Pareto-scaled principal component analysis (PCA) and orthogonal partial least-squares discriminant analysis (OPLS-DA). The 7-fold cross-validation and response permutation testing were used to evaluate the robustness of the model. The variable importance in the projection (VIP) value of each variable in the OPLS-DA model was calculated to indicate its contribution to the classification. Metabolites with a VIP value >1 and p-values < 0.05 were considered statistically significant.

References

Choi H H, Zou S, Wu J L, Wang H, Phan L, Li K, Zhang P, Chen D, Liu Q, Qin B, Nguyen T A T, Yeung S J, Fang L, Lee M H. 2020. EGF Relays Signals to COP1 and Facilitates FOXO4 Degradation to Promote Tumorigenesis. *Advanced science*, **7**, 2000681.

Cox J, Mann M. 2008. MaxQuant enables high peptide identification rates, individualized p.p.b.-range mass accuracies and proteome-wide protein quantification. *Nature biotechnology*, **26**, 1367-1372.

Fan W, Ge G, Liu Y, Wang W, Liu L, Jia Y. 2018. Proteomics integrated with metabolomics: analysis of the internal causes of nutrient changes in alfalfa at different growth stages. *BMC plant biology*, **18**, 78.

Thiellement H, Zivy M, Damerval C, Méchin V. 2007. Plant proteomics[M]. *Human Press*.

Wiśniewski J R, Zougman A, Nagaraj N, Mann M. 2009. Universal sample preparation method for proteome analysis. *Nature methods,* **6**, 359-362.

Fig. S1 Scores plots of principal components 1 (t[1]) and 2 (t[2]) of the PCA, PLS-DA, and OPLS-DA results from maize roots at different treatments.

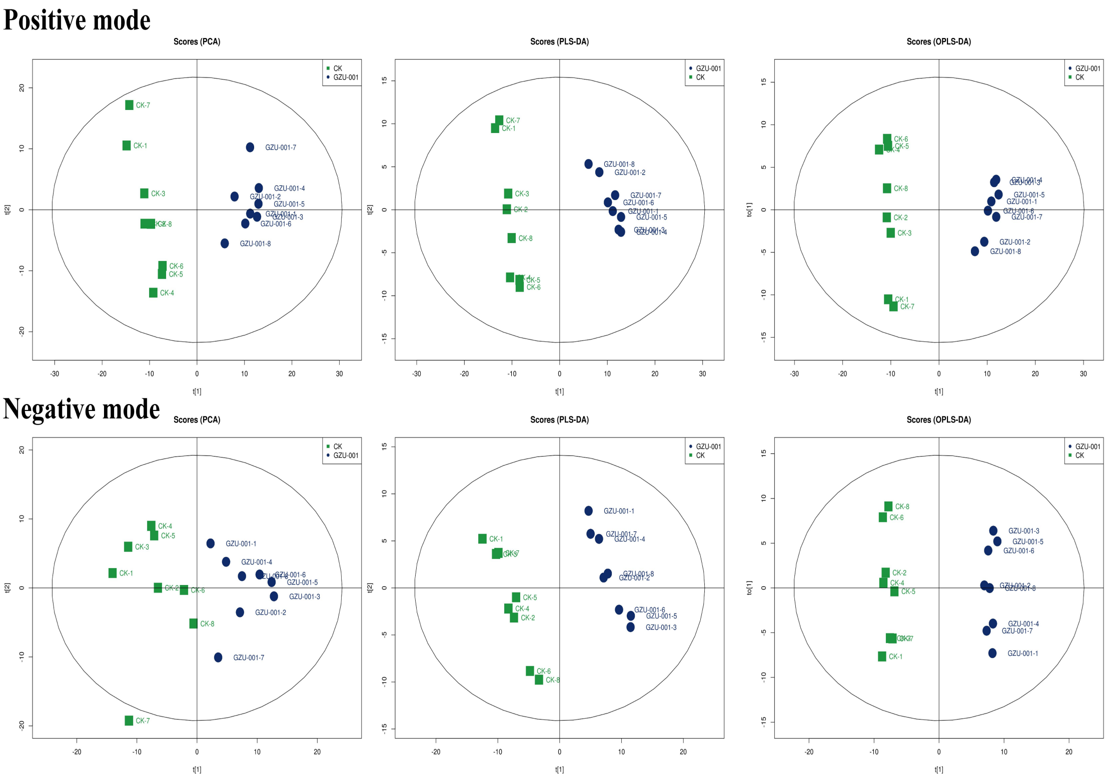

Fig. S2 Root metabolic profiles after adding GZU001. (A) Volcano plot for the differentiated metabolites in positive mode; (B) Volcano plot for the differentiated metabolites in negative mode.

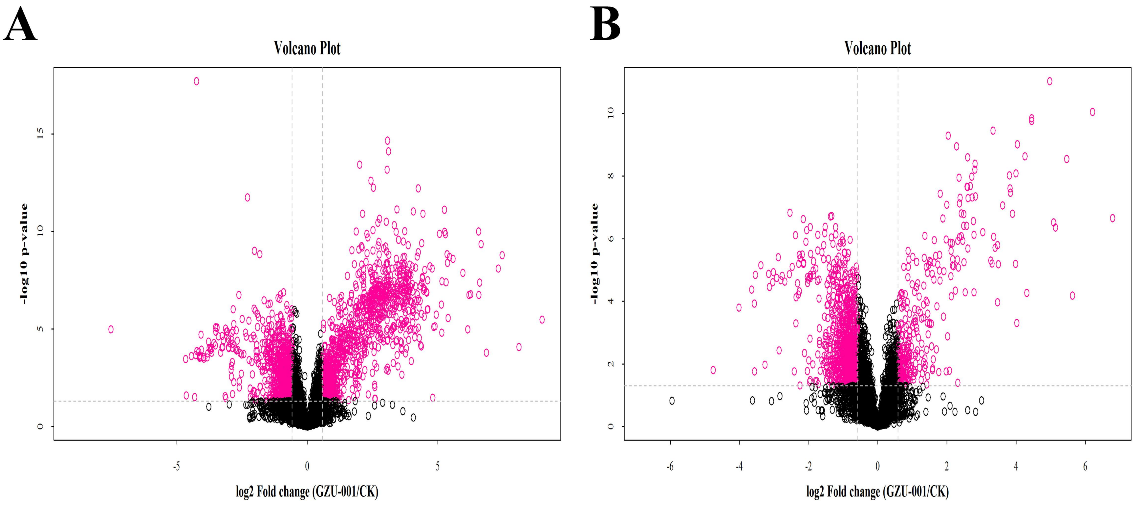

Supplement: Supplementary file 1 — Additional file 1: Fig. S1. Scores plots of principal components 1 (t[1]) and 2 (t[2]) of the PCA, PLS-DA, OPLS-DA results from maize roots at different treatment. Fig. S2. Root metabolic profiles after adding GZU001. [file 12870_2023_4130_MOESM1_ESM.pdf]
